# Resident and program characteristics that impact performance on the Ophthalmic Knowledge Assessment Program (OKAP)

**DOI:** 10.1186/s12909-019-1637-4

**Published:** 2019-06-07

**Authors:** Sidra Zafar, Xueyang Wang, Divya Srikumaran, Shameema Sikder, Pradeep Ramulu, Michael V. Boland, Eric Singman, Fasika A. Woreta

**Affiliations:** Wilmer Eye Institute, Johns Hopkins University School of Medicine, 600 North Wolfe Street, Wilmer B29, Baltimore, MD, Baltimore, MD 21287 USA

**Keywords:** Resident, Education, Knowledge, OKAP

## Abstract

**Background:**

To determine which resident and program characteristics correlate with ophthalmic knowledge, as assessed by resident Ophthalmic Knowledge Assessment Program (OKAP) performance.

**Methods:**

An online survey was sent in June 2017 to all US ophthalmology residents who took the OKAP in April 2017.

**Results:**

The survey response rate was 13.8% (192/1387 residents). The mean respondent age was 30.4 years, and 57.3% were male. The mean [SD] self-reported 2017 OKAP percentile was 61.9 [26.7]. OKAP performance was found to have a significant positive correlation with greater number of hours spent/week studying for the OKAPs (*p* = 0.007), with use of online question banks (*p* < 0.001), with review sessions and/or lectures arranged by residency programs (*p* < 0.001), and with OKAP-specific didactics (*p* = 0.002). On multivariable analysis, factors most predictive of residents scoring ≥75th percentile were, higher step 1 scores (OR = 2.48, [95% CI: 1.68–3.64, p < 0.001]), presence of incentives (OR = 2.75, [95% CI: 1.16–6.56, *p* = 0.022]), greater number of hours/week spent studying (OR = 1.09, [95% CI:1.01–1.17, *p* = 0.026]) and fewer hours spent in research 3 months prior to examination (OR = 1.08, [95% CI: 1.01–1.15, *p* = 0.020]. Lastly, residents less likely to depend on group study sessions as a learning method tended to score higher (OR = 3.40, [95% CI: 1.16–9.94, *p* = 0.026]).

**Conclusions:**

Programs wishing to improve resident OKAP scores might consider offering incentives, providing effective access to learning content e.g. online question banks, and adjusting the curriculum to highlight OKAP material. Step 1 scores may help educators identify residents who might be at risk of not performing as well on the OKAP.

**Electronic supplementary material:**

The online version of this article (10.1186/s12909-019-1637-4) contains supplementary material, which is available to authorized users.

## Background

Medical knowledge is widely accepted to be positively associated with superior care and with better patient management. More knowledgeable physicians are more likely to adhere to evidence-based guidelines in the delivery of care and achieve better patient outcomes [1–3]. Indeed, one of the key elements of residency training is to give early career physicians sufficient knowledge for carrying out their appropriate roles. The Accreditation Council for Graduate Medical Education (ACGME) lists knowledge as one of the six core competencies required for all fields in resident training. Within the field of ophthalmology, the Ophthalmic Knowledge Assessment Program (OKAP), which is a multiple-choice, in-service examination, has been used as a measure to objectively assess core ophthalmic knowledge of residents and allows for comparison among peers, programs, and prior residents. Moreover, it is meant to serve as a guide towards preparation for the American Board of Ophthalmology Written Qualifying Examination (ABO-WQE) [4]. The WQE is an important component of board certification which is typically required for credentialing.

Given the importance of physician knowledge, it is important to provide guidance for ophthalmic trainees and their programs regarding the best methods and approaches to gain the requisite knowledge. Such guidance is increasingly important given the growing myriad of resources available to residents, including the Basic and Clinical Science Course (BCSC) books compiled by the American Academy of Ophthalmology (AAO), didactics by individual programs, review courses and books, and online question banks.

This study describes a survey of ophthalmology residents across the US to determine what resident characteristics and program attributes correlate with higher levels of ophthalmic knowledge, as judged by performance on the 2017 OKAP exam.

## Methods

The study was approved by the Institutional Review Board of The Johns Hopkins University School of Medicine.

A 22-question anonymous survey (Qualtrics, Provo, UT) was emailed to all US ophthalmology program directors who were Association of University Professors of Ophthalmology (AUPO) members on June 2, 2017. The program directors were asked to forward the survey on to all residents, with response collection ending on June 30, 2017. Three reminder emails were sent during the response collection period. Program directors who forwarded the survey to their residents were asked to reply and indicate that they have done so. In the survey, participants were made aware that all data would be kept confidential and only aggregate data would be shared. Participants were informed that their completion of the survey indicated consent for participation in the study.

Data on demographics (age, sex, marital status, and year of training), geographic region of the residency training programs, and study habits (average number of months spent preparing for examination, average number of hours spent studying, average number of hours spent in research 3 month prior to exam, average work hours) were collected. Residents were asked to report their 2017 OKAP exam percentile in addition to their USMLE Step 1 scores in ranges of 10 (e.g. 210–220, 221–230, etc.). The utility of a variety of study resources was assessed with a four-point Likert scale (not at all useful, slightly useful, moderately useful, and extremely useful). The presence of program offerings such as incentives (e.g. awards, money, vacation), repercussions (e.g. remediation), and call coverage for the night prior to OKAP exam was also assessed using a 3-point scale (yes, no, and not sure). Residents’ opinion regarding the OKAP exam was examined using a 5-point Likert scale (strongly disagree, somewhat disagree, neither agree nor disagree, somewhat agree, and strongly agree).

Pearson correlation coefficients were calculated between self-reported 2017 OKAP percentile and continuous variables. The two-sample t-test was used to compare OKAP percentiles with categorical variables, and analysis of variance (ANOVA) was used for variables with more than 2 categories. Additionally, a forward stepwise multivariable logistic regression model was constructed to determine which factors were related independently to higher resident medical knowledge (scoring on the 75th percentile or higher). Variables included in the model were participant demographics (age, sex, and marital status), residency program characteristics (geographic region, use of incentives or repercussions, call coverage for the night prior to examination), USMLE step 1 scores, and examination preparation (resources used, hours spent studying, work hours and hours spent in research). A linear regression was also performed to predict OKAP percentiles using the same variables. All data were analyzed using Stata. A *p* value of ≤0.05 was considered statistically significant for all analyses.

## Results

### Participant demographics

The survey was forwarded to residents by 19 of the ophthalmology programs (*n* = 272 residents), and 192 responses were received. This represented 13.8% of the ophthalmology resident pool in 2017 (192/1387). Of the 192 participants, 183 responded with their exact percentile, while 9 could only provide ranges because they did not recall their exact percentile. All subsequent correlation analyses were completed with the 183 participants who provided their exact percentile. Participant characteristics are summarized in Table 1.

**Table 1:** Baseline participant characteristics

Demographic	
Mean age, (SD), years	30.4 (2.7)
Mean number of residents in program, (SD)	11.0 (4.6)
Mean self-reported percentile score for 2017 OKAP exam, (SD)	61.9 (26.7)
Gender	
Female, No. (%)	82 (42.7)
Male, No. (%)	110 (57.3)
Training Year	
PGY 2, No. (%)	71 (37.0)
PGY 3, No. (%)	67 (34.9)
PGY 4, No. (%)	54 (28.1)
Geographic Region of Residency Program	
Midwest, No. (%)	40 (20.8)
Northeast, No. (%)	60 (31.3)
South, No. (%)	48 (25.0)
West, No. (%)	44 (22.9)

### OKAP percentile and resident work

The mean (standard deviation [SD]) self-reported 2017 OKAP percentile from participants was 61.9 (26.7; range 4–100). The average number of hours per week spent on studying for the OKAP was significantly correlated with OKAP percentile (*p* = 0.007). OKAP percentile was not significantly correlated with program size, total number of months spent studying, average number of hours per week spent on research in the 3 months before the exam, or average number of duty hours per week logged for the ACGME (Table 2, *p* > 0.05 for all). The degree of use of online question banks (e.g., OphthoQuestions.com) was statistically significantly correlated with OKAP percentile (*p* < 0.001). Review sessions and/or lectures by programs were also correlated with OKAP percentile (*p* < 0.01). All other resources surveyed (review books, BCSC books, question books, group study sessions, and review courses) were not significantly correlated with OKAP percentile (Table 3, *p* > 0.05 for all). The write-in responses for the option of “Other” regarding study resources were “Anki cards,” “On call review for complex patients,” and “Self-made flash cards.”

**Table 2:** Resident Work and Correlation with OKAP Percentile

	Pearson’s Correlation Coefficient	*p*-value
Mean number of months spent studying for OKAP	−0.026	0.727
Mean number of hours per week spent studying for OKAP	0.201	0.007
Mean number of hours per week spent on research	−0.101	0.180
Mean number of duty hours per week logged	−0.100	0.183

**Table 3:** Correlation of different study resources utilized with OKAP Percentile

Resource	OKAP Percentile, Mean ± SD, (No. of Respondents)					*p*-Value
	Did Not Use	Not At All Useful	Slightly Useful	Moderately Useful	Extremely Useful	
Review Books (e.g. Chern, Friedman)	60.5 ± 30.6 (51)	54.3 ± 30.0 (3)	63.5 ± 26.5 (42)	63.3 ± 23.9 (55)	63.7 ± 22.4 (26)	0.946
Basic Clinical science course (BCSC) books	57.7 ± 28.3 (19)	72.5 ± 27.1 (6)	55.9 ± 27.9 (52)	63.6 ± 24.3 (62)	69.3 ± 26.2 (38)	0.122
Question Books (e.g. ProVision, Mass Eye and Ear Review)	62.3 ± 27.0 (141)	26.0 ± 31.1 (2)	66.9 ± 22.6 (14)	67.7 ± 20.8 (13)	68.5 ± 27.6 (2)	0.308
Online Question Banks (e.g. OphthoQuestions.com)	59.6 ± 25.8 (5)	N/A (0)	49.3 ± 31.4 (8)	49.9 ± 27.2 (43)	68.4 ± 23.7 (120)	< 0.001
Group Study Sessions	60.2 ± 27.6 (107)	61.5 ± 21.4 (4)	71.8 ± 27.2 (25)	63.0 ± 19.0 (28)	68.0 ± 27.1 (10)	0.359
Review Sessions/Lectures by Program	47.1 ± 28.8 (13)	48.7 ± 28.8 (9)	55.4 ± 27.1 (65)	67.7 ± 24.0 (63)	77.7 ± 18.8 (26)	< 0.001
Review Courses (e.g. Wills Eye, Osler, San Antonio)	63.6 ± 26.4 (128)	48.0 ± 11.5 (3)	60.8 ± 28.5 (14)	58.6 ± 24.1 (18)	66.3 ± 28.8 (12)	0.770

### OKAP percentile and program characteristics

When residents were asked how much they agree that their year-round didactics by their residency program emphasized OKAP material, those who strongly agreed with the statement had significantly higher OKAP scores than those who strongly disagreed (mean OKAP percentile 65.8 versus 37.8, *p* = 0.002). Residents’ opinion on whether their OKAP performance was important to their residency program or fellowship applications did not correlate with their 2017 OKAP performance (Table 4).

**Table 4:** Correlation between resident opinion on OKAP-specific statements and their OKAP percentile

Statement	OKAP Percentile, Mean ± SD (No. of Respondents)					*p*-value
	Strongly Disagree	Somewhat Disagree	Neither Agree Nor Disagree	Somewhat Agree	Strongly Agree	
My residency program places importance on my OKAP performance	61.3 ± 38.5 (4)	45.8 ± 27.4 (12)	56.3 ± 23.9 (42)	60.8 ± 27.3 (81)	67.7 ± 24.1 (69)	0.075
My OKAP performance is important for fellowship applications	61.4 ± 31.4 (9)	59.0 ± 32.3 (25)	62.4 ± 23.5 (43)	60.8 ± 26.6 (81)	71.7 ± 21.9 (20)	0.537
My year round didactics emphasize OKAP material	37.8 ± 27.7 (15)	57.2 ± 28.9 (31)	64.5 ± 26.6 (34)	66.9 ± 24.3 (76)	65.8 ± 20.0 (20)	0.002

Furthermore, residents were asked whether their programs offered incentives for high OKAP performance, repercussions for poor OKAP performance, and/or call coverage for the night prior to OKAP exam. Those whose programs offered incentives had significantly higher OKAP scores than those whose programs did not (mean OKAP percentile 72.9 versus 57.6, *p* = 0.003). Residents whose programs had implemented repercussions for poor scores did not perform significantly differently from residents whose programs did not (mean OKAP percentile 59.7 versus 59.4, *p* = 0.98). OKAP performance also did not differ for residents whose programs had call coverage for the night before compared to those whose programs did not (mean OKAP percentile 63.2 versus 56.3, *p* = 0.59).

### Regression analysis

Results of the univariable analysis are shown in Table 5. On multivariable analysis, factors that were most predictive of higher ophthalmic knowledge as predicted by residents scoring on the 75th percentile or higher were higher step 1 scores (defined as every 10-point increase in scores) (odds ratio (OR) = 2.48, [95% confidence interval (CI): 1.68–3.64, *p* < 0.001), presence of incentives (OR = 2.75, [95% CI: 1.16–6.6.56, *p* = 0.022]), spending more hours on average per week studying (OR = 1.09, [95% CI:1.01–1.17, *p* = 0.026]) and spending fewer hours in research three months prior to examination (OR = 1.08, [95% CI: 1.01–1.15, *p* = 0.020]. Lastly, residents less likely to depend on group study sessions as a learning method tended to score higher (OR = 3.40, [95% CI: 1.16–9.94, *p* = 0.026]) (Table 6). Higher Step 1 scores were associated with ophthalmology residents being less likely to score below the 30th percentile on their the OKAP examinations (Additional file 1: Table S1).

**Table 5:** Univariable regression analysis of factors predictive for scoring above the 75th percentile on the OKAPS

Variable	Odds Ratio	95% Confidence interval	*P*-value
Age	0.90	0.80–1.01	0.075
Sex (reference female)			
Male	1.89	1.04–3.45	0.037
Marital status (reference married)			
Single	1.01	0.55–1.86	0.972
Training year (reference PGY-2)			
PGY-3	0.90	0.45–1.78	0.755
PGY-4	0.95	0.46–1.95	0.886
Geographic region (reference Midwest)			
Northeast	1.42	0.61–3.30	0.410
South	0.91	0.36–2.62	0.837
West	3.00	1.23–7.33	0.016
Incentives (reference no)			
Yes	2.40	1.27–4.54	0.007
Repercussions (reference no)			
Yes	1.05	0.52–2.10	0.896
Call coverage prior to OKAP (reference no)			
Yes	4.23	0.50–35.86	0.186
Review books (reference did not use)			
Not very useful	1.28	0.58–2.82	0.540
Extremely/moderately useful	0.87	0.43–1.75	0.691
Basic Clinical science course (BCSC) books (reference did not use)			
Not very useful	1.24	0.44–3.54	0.683
Extremely/moderately useful	1.55	0.58–4.16	0.383
Question books (reference did not use)			
Not very useful	1.50	0.56–4.00	0.418
Extremely/moderately useful	1.17	0.41–3.31	0.772
Online question banks (reference did not use)			
Not very useful	2.40	0.18–32.88	0.660
Extremely/moderately useful	2.87	0.31–26.21	0.930
Group study sessions (reference did not use)			
Not very useful	2.48	1.11–5.56	0.027
Extremely/moderately useful	1.13	0.54–2.38	0.741

**Table 6:** Multivariable regression analysis of factors predictive for scoring above the 75th percentile on the OKAPS

Variable	Odds Ratio	95% Confidence interval	*P*-value
Sex (reference female)			
Male	2.14	0.94–4.90	0.070
Region (reference Midwest)			
Northeast	3.49	0.97–12.56	0.056
West	3.67	0.99–13.50	0.051
South	2.90	0.74–11.45	0.128
Lectures (reference did not use)			
Not very useful	0.60	0.10–3.61	0.578
Extremely/moderately useful	1.42	0.13–4.85	0.795
Group study (reference did not use)			
Not very useful	3.40	1.16–9.94	0.026
Extremely/moderately useful	0.67	0.53–3.78	0.481
Incentives (reference no)			
Yes	2.75	1.16–6.56	0.022
Average number of hours per week spent studying	1.09	1.01–1.17	0.026
Average number of hours spent in research 3 months prior to exam	0.93	0.87–0.99	0.020
Step 1	2.48	1.68–3.64	< 0.001

OKAP percentile was also evaluated as a continuous variable. We found OKAP percentile to significantly correlate with incentive-based performance (beta =13.72, [95%CI: 6.74, 20.70; *p* < 0.001)], Step 1 scores (beta = 8.56, [95% CI: 5.99, 11.13; *p* < 0.001)], and male gender (beta = 7.79, [95% CI: 1.24, 14.34; *p* = 0.020)]. Residents who found review sessions and lectures offered by their programs to have limited value were more likely to score lower (beta = − 8.18, [95% CI: − 15.19, − 1.17; *p* = 0.022)] compared to those who found them to be useful. Spending greater than 20 h per week in research was also negatively associated with OKAP performance (beta = − 17.42, [95% CI: − 2.45, − 32.39; *p* = 0.023)].

## Discussion

In our study, we found resident OKAP performance, and by extension, ophthalmic knowledge, to significantly correlate with more hours per week spent studying as well as with incentive-based performance. Among educational resources, year-round didactics, online question banks, and review sessions were associated with better OKAP performance. To the best of our knowledge, resident ophthalmic knowledge has been evaluated in only 1 other study. The authors of that study found an association between resident knowledge, completion of clinical rotations and learning via multiple modalities. However, their study assessed resident knowledge specifically on the topic of glaucoma [5].

The results of our study have important implications for educators. In addition to being the only tool for assessing overall program effectiveness [6], multiple studies have shown an association between OKAP performance and the ABO-WQE pass rate [6–8]. Board certification has historically been considered a sign of physician competence. In many cases, it is a prerequisite for employment and academic appointment [6] and certification status has been shown to be an important predictor of patient quality of care among medical professionals [9–11]. Johnson et al. reported a 5.43-fold increased odds of passing the WQE among residents passing the OKAP examinations, (defined as overall score in the 30th percentile or above by the authors), in all 3 years of residency training. In contrast, failing all 3 OKAP examinations was associated with more than 9-fold lower odds of passing the WQE on the first attempt [5]. Similarly, a separate study by Lee et al. that involved 246 residents from 15 institutes found OKAP scores across all 3 years of residency to be predictive of first time WQE pass rate, with third year OKAP scores being the most predictive. Additionally, the authors found a correlation between the mean OKAP scores and WQE pass rate; passing the WQE was at least 80% for a score of 35 or higher, at least 90% for a score of 53 or higher, and at least 95% for a score of 72 or higher [6]. Furthermore, ophthalmic knowledge may have important implications for a graduating resident’s career, since OKAP performance is one of the factors currently used in the sub-specialty fellowship selection process [12]. As the OKAP is currently one of our best assessments of ophthalmic knowledge and because it serves as an important predictor of WQE performance, it is essential that residency programs place emphasis on improving study practices.

In our study, spending greater than 20 h per week in research-related activities closer to the scheduled examination was found to negatively affect resident OKAP performance. We believe that these residents likely preferred spending more time on research-related activities than studying for OKAPs. Furthermore, resident training years are commonly associated with long and unpredictable work hours. Physician burnout and emotional exhaustion have in fact, been shown to result in decreased medical knowledge [13, 14]. The challenge for medical education is therefore, to maintain knowledge while concurrently ensuring physician well-being and adequate patient safety. There is no question that the demands of surgical residency sometimes make reading and studying difficult. Possible changes can include allocating more time for resident self-study, offering review sessions which are tailored to meet the needs of residents and providing effective access to learning content. Residents may also consider maintaining a regular study schedule throughout the year, which has been shown to result in improved medical knowledge and better performance on standardized examinations [14, 15]. Although we did not assess the effectiveness of electronic or E-learning in our study, residency programs could also consider complementing traditional didactic methods with E-learning. E-learning has been shown to be as effective as other educational approaches for acquisition of knowledge [16]. Additionally, the greater flexibility offered by E-learning courses could make them particularly useful for graduate medical education [17]. We hypothesize that improved practices such as these will result in successful acquisition and retention of knowledge among residents. This is important because knowledge is currently one of the six core competencies that graduating residents are required to achieve competency in. [18]The OKAP can therefore be used as a benchmark for ophthalmology program effectiveness in medical knowledge [18] and additionally, as an educational tool in meeting the residency training requirement for board certification [19].

While increased emphasis of program policies towards in-training examinations as well as improved resident studying habits have been known to correlate with better knowledge acquisition [14, 20–22], limited data currently exist on how this knowledge translates into clinical performance in the real world. A cross-sectional study conducted by Catalano et al. failed to demonstrate any relationship between OKAP scores and resident clinical performance [23] and a separate study by Scott et al. similarly failed to show an association between American Board of Surgery In-Training Examination (ABSITE) scores, technical skill and operative performance [24]. This lack of association however, may have been due to several different factors, including a small sample size [23, 24] and that clinical performance was judged subjectively [23]. Future studies are needed to assess whether resident knowledge levels are associated with other competency milestones such as patient care and procedural skills.

USMLE Step 1 scores are widely regarded to be a valid measure of underlying medical knowledge [25] and our study results are consistent with the notion that, medical students who are most successful with step 1 of the USMLE will be more able to acquire the knowledge needed to satisfactorily complete their ophthalmology training. We observed almost a 9-point increase in the OKAP percentile and 2.5 times higher odds of scoring above the 75th percentile, when USMLE scores moved up by every 10-point category. As such, this information benefits educators by allowing them to identify residents at risk of performing poorly, as early as possible. Identifying residents with insufficient medical knowledge is important for several reasons. Most importantly, physicians who lack adequate medical knowledge may be more likely to make errors in diagnosis and perform less well clinically, which can jeopardize patient safety [13, 26].

Interestingly, we found residents who were less likely to rely on group study for learning, performed better. A study involving 45 general surgery residents reported similar findings, with independent study methods identified as the most effective study method for successful American Board of Surgery examination performance [27]. Potential reasons for this include that, residents partaking in self-study may have greater focus and fewer distractions as well as the ability to study at one’s own pace while identifying areas of weakness that require greater reinforcement. Self-study may also be associated with higher levels of concentration, which has been shown to be an important predictor for learning [28]. However, it is important to approach these findings of group versus self-study with caution. It might be that respondents who preferred self-study generally do well on standardized tests. Furthermore, it might be that the responses we received might have been skewed from respondents who preferred self-study over group study. Incentive-based performance was found to be a very important motivator for medical knowledge acquisition in our study sample. Ophthalmic knowledge, as indicated by the OKAP scores, increased by 15 points for programs that offered incentives in the form of awards, money or vacation, compared to those that did not. Residents belonging to such programs were 3-times more likely to be in the top 25th percentile for medical knowledge concerning their field when compared with their peers. In contrast, no such relation was seen when repercussions were employed by residency programs for poor OKAP performance. Similar findings regarding incentive use and improved test performance have also been reported among school-going children [29]. In medicine, incentives have been used to improve trainee compliance with patient safety measures [30] and encourage faculty productivity in educational activities [31]. Furthermore, the recently introduced Merit-Based Incentive Payment System rewards U.S. physicians practicing higher-value care with higher fees [32]. Thus, by incentivizing examinations, residency programs might influence resident motivation and thereby improve knowledge levels.

One major limitation to our study was the limited response rate. Although we sent the survey to all training programs in the country, only 19 (15.7%) programs forwarded the survey to their residents. However, our program and respondent characteristics are comparable to that of the U.S. ophthalmology residency programs as well as the ophthalmology resident population. Based on our analysis of 121 U.S. ophthalmology residency programs, we found the average program size to be 12 residents, with the following regional distribution: Midwest 23.1%, Northeast 30.6%, South 28.9% and West 17.4%. Similarly, 240–250 was the most common Step 1 score range among our residents, which is again comparable to the average Step 1 score of 242–245 as reported by the San Francisco Match (SF Match) for the years 2014–2018. Furthermore, our study response rate, despite being at the lower end of the spectrum, is within the response rate range of surveys (10–51%) conducted in the field of ophthalmology [33–35]. Given our limited response rate, it would be beneficial to assess our results using a large multi-center study, especially for understanding factors associated with residents scoring less than the 30th percentile. Secondly, the distribution of self-reported OKAP percentile is positively skewed, indicating sampling bias, as those who scored higher on the OKAP might be more willing to complete the survey. Since residents were asked by report their OKAP percentiles, recall bias may be present, although the survey was distributed shortly after the 2017 OKAP results were released. Future studies assessing exact individual scores reported to programs (as opposed to self-reported) and correlations with objective clinical performance may be useful.

## Conclusion

In conclusion, our study demonstrates several factors that correlate with OKAP performance and ways programs may strengthen resident medical knowledge as measured by OKAP performance. Programs wishing to improve resident learning and education might consider implementing strategies designed to increase knowledge acquisition. Strategies supported by our results include implementing incentives and effective access to learning content, with scheduling didactics dedicated to OKAP material and making online question banks available to residents. It may be worthwhile to consider allocating more time for resident self-study. Furthermore, Step 1 scores can help educators identify early on, residents who might be at risk for attaining insufficient medical knowledge and, for not performing as well on the OKAP. Future studies are needed to investigate whether improved resident knowledge, predicts clinical acumen after training or the Oral Exam pass rate, which is administered after the successful completion of the WQE and whether medical knowledge is correlated with other ACGME-mandated competencies.

## Additional file

Additional file 1: **Table S1A.** Factors associated with participants scoring less than 30th percentile on the OKAP examination. (DOCX 18 kb)

## Data Availability

Not applicable.

## Abbreviations

AAO: American Academy of Ophthalmology
ABO-WQE: American Board of Ophthalmology Written Qualifying Examination
ABSITE: American Board of Surgery In-Training Examination
ACGME: Accreditation Council for Graduate Medical Education
ANOVA: Analysis of Variance
AUPO: Association of University Professors of Ophthalmology
BCSC: Basic and Clinical Science Course
OKAP: Ophthalmic Knowledge Assessment Program
SF Match: San Francisco Match
