# Supplementary material for: Resident and program characteristics that impact performance on the Ophthalmic Knowledge Assessment Program (OKAP)
Source: BMC Med Educ. 2019 Jun 7;19:190. doi: 10.1186/s12909-019-1637-4 (PMC6555746; doi:10.1186/s12909-019-1637-4)
Supplement: Supplementary file 1 — Table S1A. Factors associated with participants scoring less than 30th percentile on the OKAP examination. (DOCX 18 kb) [file 12909_2019_1637_MOESM1_ESM.docx]

| **Univariate analysis** | | |
| --- | --- | --- |
| **OKAP score <30^th^ percentile** | **OR (95% CI)** | **P-value** |
| Age | 1.04 (0.90-1.21) | 0.593 |
| Gender (reference female)  Male | 0.64 (0.29-1.42) | 0.272 |
| Training year (reference PGY2)  PGY3  PGY4 | 0.30 (0.10-0.88)  0.73 (0.29-1.83) | 0.028  0.501 |
| Geographic region |  |  |
| Incentives (reference no)  Yes | 0.23 (0.07-0.81) | 0.022 |
| Repercussions (reference no)  Yes | 1.00 (0.41-2.46) | 0.995 |
| Call coverage |  |  |
| Review books (reference did not use)  Not very useful  Moderately/extremely useful | 0.57 (0.19-1.71)  0.52 (0.20-1.33) | 0.318  0.172 |
| Question books (reference did not use)  Not very useful  Moderately/extremely useful | 0.75 (0.16-3.54)  Empty | 0.716 |
| Online question banks (reference did not use)  Not very useful  Moderately/extremely useful | 3.43 (0.77-15.28)  Empty | 0.129 |
| Group study (reference did not use)  Not very useful  Moderately/extremely useful | 0.78 (0.24-2.52)  0.59 (0.19-1.88) | 0.681  0.375 |
| Lectures (reference did not use)  Not very useful  Moderately/extremely useful | 1.30 (0.34-4.97)  1.26 (0.43-3.73) | 0.704  0.675 |
| Number of hours per week spent studying | 0.84 (0.39-1.80) | 0.653 |
| Number of months spent studying | 1.15 (0.67-2.00) | 0.610 |
| Number of hours per week spent on research | 1.71 (0.89-3.29) | 0.105 |
| Number of hours per week spent working | 1.02 (0.42-2.48) | 0.964 |
| Step 1 score | 0.48 (0.33-0.69) | <0.001 |
| **Multivariate analysis** | | |

**Additional file 1: Table S1A:** Factors associated with participants scoring less than 30^th^ percentile on the OKAP examination

| **Failed OKAP**  **(<30^th^ percentile)** | **OR (95% CI)** | **P-value** |
| --- | --- | --- |
| Gender (reference female)  Male | 0.68 (0.25-1.84) | 0.449 |
| Training year (reference PGY2)  PGY3  PGY4 | 0.27 (0.08-0.95)  0.53 (0.16-1.74) | 0.041  0.294 |
| Incentives (reference no)  Yes | 0.32 (0.08-1.26) | 0.104 |
| Online question banks (reference did not use)  Not very useful  Moderately/extremely useful | 2.02 (0.26-15.6)  Empty | 0.501 |
| Number of hours per week spent on research | 1.72 (0.72-4.13) | 0.222 |
| Step 1 Score | 0.42 (0.28-0.64) | <0.001 |
